# Hypervigilance and kinesiophobia characterize distinct exploratory data-driven profiles of temporomandibular disorders

**DOI:** 10.1038/s41598-026-60271-w

**Published:** 2026-06-29

**Authors:** Adriana Battisti Archer, Jonas Davi da Silva Mello Noel, Javier Salinas, Diego De Nordenflycht, Pedro Miguel Teixeira Carvas Cebola, Tassia Tillemont Machado, Ricardo de Souza Tesch, Thayanne Brasil Barbosa Calcia, Beatriz Dulcineia Mendes de Souza, Gilberto Melo, Markus Barhanko, Anastasios Grigoriadis, Dyanne Medina Flores, Giancarlo De la Torre Canales

**Affiliations:** 1 Department of Dentistry, Federal University of Santa Catarina (UFSC), Florianópolis, Brazil; 2 Chronic Pain Research Group, Centro Universitário Arthur Sá Earp Neto (UNIFASE), Petrópolis, Brazil; 3 Faculty of Dentistry, Universidad Andres Bello, Viña del Mar, Chile; 4 Orofacial Pain Unit, CUF Tejo Hospital, Lisboa, Portugal; 5 Egas Moniz Center for Interdisciplinary Research (CiiEM), Egas Moniz School of Health & Science, Caparica, Almada Portugal; 6 Department of Functional and Structural Biology, Institute of Biology - Pain Studies Laboratory, State University of Campinas (UNICAMP), Campinas, Brazil; 7 Department of Dental Medicine, Karolinska Institutet, Huddinge, Sweden

**Keywords:** Hypervigilance, Kinesiophobia, Temporomandibular joint disorders, Phenotype, Diseases, Health care, Medical research, Psychology, Psychology, Risk factors, Signs and symptoms

## Abstract

Hypervigilance and kinesiophobia have been associated with temporomandibular disorders (TMD), but their relationship with specific diagnostic profiles remains unclear. This study investigated the association between TMD diagnoses, hypervigilance, and kinesiophobia, and whether these variables could differentiate distinct clinical profiles across painful and non-painful conditions. In this multicenter cross-sectional observational study, 862 adults aged 18 to 50 years from Brazil, Chile, and Portugal were classified as controls, non-painful TMD, or painful TMD. Diagnoses were established using the Diagnostic Criteria for Temporomandibular Disorders (DC/TMD). Hypervigilance and kinesiophobia were assessed using the Pain Vigilance and Awareness Questionnaire (PVAQ) and the Tampa Scale for Kinesiophobia for TMD (TSK/TMD). Statistical analyses included factor analysis of mixed data, hierarchical clustering on principal components, and partial least squares discriminant analysis, followed by multivariate and regression analyses to assess potential associations between psychosocial variables and TMD diagnoses. Among 862 participants (312 controls, 550 TMD), kinesiophobia and hypervigilance increased across groups, with the highest levels in painful TMD (*p* < 0.001). Unsupervised clustering identified three distinct profiles aligned with clinical presentation: controls with low psychological burden, non-painful TMD with intermediate levels, and painful TMD with elevated kinesiophobia and hypervigilance. Multivariate analyses showed associations of psychosocial variables with TMD diagnoses, with kinesiophobia consistently driving group differences, while age, gender, and pain vigilance had a moderate influence. Among TMD subgroups, joint pain was associated with higher pain vigilance and muscular TMD with higher kinesiophobia, suggesting greater clinical complexity. Higher levels of hypervigilance and kinesiophobia were associated with painful and clinical more complex TMD profiles.

**Supplementary Information:**

The online version contains supplementary material available at 10.1038/s41598-026-60271-w.

## Introduction

Chronic pain conditions are increasingly understood within a biopsychosocial framework in which biological, psychological, and behavioral processes interact to shape the onset, perception, and persistence of pain^1,2^. Beyond peripheral nociceptive mechanisms, cognitive, behavioral and affective factors can influence how pain signals are interpreted, attended to, and behaviorally managed^1,2^. Experimental and clinical studies have shown that cognitive-behavioral process such as pain-related attention (hypervigilance), fear of movement (kinesiophobia), and maladaptive beliefs (catastrophization) can amplify pain perception, contribute to disability, and affect clinical outcomes across several chronic pain conditions, including temporomandibular disorders (TMD)^3–6^.

Hypervigilance refers to a state of increased and sustained attentional focus on bodily sensations perceived as potentially threatening, including pain-related signals^7,8^. In individuals with chronic pain, this attentional bias may lead to increased monitoring of somatic sensations and difficulty disengaging attention from painful stimuli and the affected area^3,7^. Experimental research suggests that such attentional processes may enhance the salience of nociceptive input and contribute to the persistence of pain by reinforcing symptom awareness and concern^3,7^. Kinesiophobia, in contrast, refers to an excessive fear of movement arising from the expectation that physical activity may cause or worsen pain or injury^9,10^. This construct is closely linked to the fear-avoidance model of chronic pain, which proposes that individuals who interpret pain as threatening may develop fear-related responses that promote avoidance behaviors^10,11^. Over time, reduced movement and activity may lead to functional limitation, deconditioning, and maintenance of pain^10,11^.

In the context of TMD (a group of conditions affecting the temporomandibular joint, the masticatory muscles, and associated anatomical structures), evidence indicates that cognitive-behavioral variables like kinesiophobia and hypervigilance may contribute to pain-related responses and differences in symptom expression^12–14^. Studies have reported higher levels of kinesiophobia in individuals with TMD compared with healthy controls, particularly in those with painful TMD diagnosis and higher number of TMD diagnoses, and associations of kinesiophobia with pain intensity, disability, functional impairment and clinical complexity^9,12,15,16^. Compared with kinesiophobia, hypervigilance has been less extensively investigated in TMD, although emerging evidence suggests associations with TMD and kinesiophobia^13^. Taken together, these findings suggest that both variables may be relevant to the clinical expression of TMD^12,13,16^. However, their combined relationship with specific TMD diagnosis and complex TMD clinical presentations remains insufficiently understood^12,13^. Moreover, most previous investigations have relied on conventional correlation or regression-based approaches, which may not fully capture the multidimensional interactions between psychological and clinical physical variables. Considering the heterogeneity of TMD, approaches based on multivariate and data drive profiling may provide a more comprehensive characterization of distinct clinical presentations. In particular, unsupervised clustering analysis may help identify subgroups sharing similar psychosocial and pain-related patterns beyond traditional diagnostic classifications.

Understanding how these cognitive-behavioral variables are associated with different clinical presentations may contribute to a more comprehensive characterization of the disorder. Therefore, this study aimed to investigate the association of kinesiophobia, hypervigilance, and TMD diagnoses in a multicenter sample. Additionally, this study explored whether cluster-based and multivariate analyses could identify different patterns of psychosocial burden with painful and non-painful TMD conditions.

## Methods

### Study design and ethical considerations

A multicenter cross-sectional observational study was conducted including participants from three countries (Brazil, Chile, and Portugal). Data collection was carried out between December 2023 and June 2025 at the Faculty of Dentistry of Universidad Andrés Bello (Viña del Mar, Chile), the Egas Moniz Dental Clinic (Almada, Portugal), the CUF Tejo Hospital (Lisbon, Portugal), and Centro Universitário Arthur Sá Earp Neto (UNIFASE, Petrópolis, Rio de Janeiro, Brazil). Participants were recruited among individuals seeking routine dental care or evaluation for TMD. The study protocol was approved by the respective institutional research ethics committees of Andres Bello University, Chile: #56-2024; Egas Moniz School of Health and Science, Portugal: #1273; CUF TEJO Hospital, Portugal: #33,956,737/2023 and Centro Universitário Arthur Sá Earp Neto (UNIFASE), Brazil: 6.826.183/2024 and conducted in accordance with the principles of the Declaration of Helsinki. All participants received detailed information about the study procedures and provided written informed consent prior to enrollment. They were also informed of their right to withdraw from the study at any time without any consequences. The reporting of the study adhered to the Strengthening the Reporting of Observational Studies in Epidemiology (STROBE) guidelines^17^.

### Participants

Adult individuals aged between 18 and 50 years were invited to participate. Recruitment included both individuals diagnosed with TMD and individuals without TMD symptoms who served as controls. Before the clinical assessment, participants underwent a pre-screening questionnaire to evaluate eligibility criteria. Individuals were not included if they reported undergoing treatment for orofacial pain, previous treatment for TMD, ongoing orthodontic therapy, a history of significant rheumatologic, neurological, or psychiatric conditions that could interfere with pain perception or questionnaire responses or had diagnoses of other orofacial pain conditions besides TMD. Participants were evaluated through a standardized clinical examination based on the Diagnostic Criteria for Temporomandibular Disorders (DC/TMD)^14^. The assessments were performed by trained examiners with prior experience in the application of the DC/TMD protocol.

### Study protocol

All participants underwent a single evaluation session. The initial screening was performed using the TMD Screener (sensitivity of 99% and specificity of 97% for identifying true positive and true negative cases) incorporated in the DC/TMD^17^. Then, participants received a comprehensive clinical assessment based on the full DC/TMD protocol and based on the findings were classified as TMD or controls. Then, participants were informed about the study procedures and completed the self-report questionnaires used in the study, including the Pain Vigilance and Awareness Questionnaire (PVAQ) and Tampa Scale for Kinesiophobia/TMD (TSK) Questionnaire^9,19–24^.

### Outcomes

#### TMD diagnosis

TMD diagnoses were established using the DC/TMD^14^. For analytical purposes, TMD status was operationalized in several ways. First, participants were classified into two categories according to the presence or absence of TMD. Second, participants were categorized based on the presence of painful TMD conditions into three groups: painful TMD, non-painful TMD, and controls. Finally, TMD diagnostic subgroups included arthralgia, joint disorders, myalgia (local myalgia, myofascial pain, myofascial pain with referral and headache attributed to TMD) and the combinations of these subgroups (painful diagnoses, arthralgia + joint disorders, myalgia + joint disorders and painful diagnosis + joint disorders).

#### Tampa Scale for Kinesiophobia/TMD (TSK) Questionnaire

Fear of jaw movement was assessed using the validated Spanish and Portuguese versions of the Tampa Scale for Kinesiophobia for Temporomandibular Disorders (TSK/TMD), a self-reported questionnaire specifically developed to evaluate movement-related fear and avoidance behaviors associated with TMD^9,23,24^. The instrument consists of items rated on a 4-point Likert scale (from “1 = strongly disagree” to “4 = strongly agree”), and the overall score corresponds to the sum of individual responses. Higher values represent meaningful levels of TMD-kinesiophobia. Its psychometric properties demonstrate good reliability (> 0.75) and construct validity, supported by factor analysis and associations with related psychosocial measures^23,24^.

#### Pain Vigilance and Awareness Questionnaire (PVAQ)

Pain-related attention and monitoring were measured using the validated Spanish and Portuguese version of the Pain Vigilance and Awareness Questionnaire (PVAQ)^19,20,22,25^. This self-administered instrument evaluates the extent to which individuals focus on and monitor pain-related sensations in daily life. The questionnaire includes items rated on a 6-point scale (from 0 = never to 5 = always), and the total score is calculated by summing all responses. Higher scores indicate greater levels of hypervigilance to pain. Its psychometric properties demonstrate good reliability (> 0.85) and construct validity, supported by factor analysis and associations with pain-related psychological measures^19,20,22,26^.

### Statistical analysis

Qualitative variables were described using absolute and relative frequencies, whilst quantitative variables were summarized using means and standard deviations (SD). All statistical analyses were conducted using R (version 4.5.3; R Foundation for Statistical Computing, Vienna, Austria) using the following main packages: FactoMineR, factoextra, MANOVA.RM, mvabund, performance, psych, ropls, and rstatix. Outliers were evaluated using Mahalanobis distance, and observations exceeding the critical chi-square threshold (*p* < 0.001) were examined and excluded. Statistical analyses were conducted with the variables age, gender, TMD status, PVAQ, and TSK/TMD scores, with significance level set at *p* < 0.05. The only variable excluded from statistical analyses was participants’ country of origin, as it was considered a contextual geographic variable rather than a measurable psychosocial attribute.

As an initial step to reduce data dimensionality and identify underlying patterns across variables (age, gender, PVAQ, and TSK/TMD scores), a factor analysis of mixed data was conducted. The number of retained dimensions was determined according to the Kaiser criterion (eigenvalues ≥ 1.0), supplemented by scree plot inspection.

Given the multidimensional nature of the clinical data, an exploratory cluster analysis was performed to assess whether data-driven groupings aligned with the predefined diagnostic categories. Unsupervised hierarchical clustering on principal components was applied to the factor scores of the retained factor analysis dimensions, reducing noise compared to raw data. Ward’s D2 linkage method was used, followed by k-means consolidation to refine cluster assignments. The optimal number of clusters was determined based on the relative loss of inertia between successive partitions, supported by dendrogram inspection. Cluster stability was evaluated through bootstrap resampling (*n* = 1000) using Jaccard similarity coefficients, with values ≥ 0.75 considered indicative of stable clusters. Internal validation of the cluster solutions was performed using silhouette analysis, with average silhouette width interpreted as follows: no substantial structure (≤ 0.25), weak (0.26–0.50), moderate (0.51–0.70), and strong (≥ 0.71). Standardized v-test statistics were used to characterize the contribution of qualitative variables to each cluster, while eta² values were used to estimate the effect sizes of quantitative variables.

To further explore group structure and variable contributions to group discrimination, a supervised analysis was performed using partial least squares discriminant analysis. Variables were centered and scaled prior to modelling and variable importance in projection (VIP) scores were calculated to identify variables contributing to group discrimination, with VIP ≥ 1 used as the threshold for meaningful contribution to group discrimination.

Considering that clusters mostly aligned with the diagnostic groups, the diagnostic classification was retained for all subsequent analyses, given its greater clinical interpretability. In this context, TSK/TMD and PVAQ scores were compared across diagnostic groups using multivariate analysis of covariance (MANCOVA) adjusting for age and gender, with Type III sums of squares, sum-to-zero contrasts, and mean-centred age. Since normality (Mardia’s; Shapiro–Wilk) and covariance homogeneity (Box’s M) were violated, Pillai’s trace was used, with the F-tests remaining robust to non-normality given the large sample size (central limit theorem). As the homogeneity of slopes assumption was violated for age, a diagnostic group × age interaction was included; a diagnostic group × gender interaction was tested but non-significant, therefore not included in the final model. Significant multivariate effects were followed by univariate Type III ANCOVAs, with Holm-adjusted pairwise comparisons of estimated marginal means between diagnostic groups. Significant group × age interactions were decomposed using simple slopes and Johnson–Neyman regions of significance.

In a separate analysis conducted exclusively among TMD-only subset participants, to evaluate multivariate associations between psychosocial factors (age, gender, PVAQ, and TSK/TMD scores) and diagnostic subgroups that may co-occur within the same individual (joint pain, joint disorder, and muscular TMD), a multivariate generalized linear model (manyglm) was constructed. A binomial distribution was specified to account for the binary nature of each subgroup (absence vs. presence), and significance was assessed via Wald statistics using PIT-trap resampling (1,000 iterations), which accounts for correlations between outcome variables. Follow-up univariate Wald tests were subsequently conducted to identify outcome-specific associations, with p-values adjusted for multiple comparisons via step-down resampling.

Lastly, to assess whether psychosocial factors were associated with the likelihood of a TMD diagnosis, a multinomial logistic regression was conducted. The dependent variable was diagnostic group (control, non-painful TMD, Painful TMD), with control set as the reference category. Independent variables included age, gender, TSK/TMD scores, and PVAQ scores, which were entered simultaneously. The relative odds ratio to each TMD group versus the control group were estimated. Multicollinearity was assessed via variance inflation factors (all VIF < 1.35), indicating no major concerns. Classification performance was assessed per category using the one-vs-rest area under the receiver operating characteristic curve (AUC), whilst the accuracy of the predicted probabilities was evaluated via the Brier score.

## Results

### Sample characteristics and psychosocial status

During the screening phase, 175 individuals were excluded across the three participating countries based on the predefined eligibility criteria, corresponding to 16.9% of all individuals assessed for eligibility. The final sample consisted of 862 individuals, including 312 (36.2%) controls and 550 (63.8%) diagnosed with TMD (non-painful TMD = 160 (18.6%) and painful TMD = 390 (45.24%). Females predominated across all groups, particularly among participants with non-painful TMD (66.3%) and painful TMD (77.9%). Participants were recruited from three countries: Brazil (*n* = 215), Chile (*n* = 355), and Portugal (*n* = 292). Descriptive data on gender distribution, age, PVAQ, and TSK/TMD scores across the three diagnostic groups are presented in Table 1.

**Table 1:** Descriptive characteristics across TMD diagnostic groups.

Variables	Diagnostic groups		
	Control (*n* = 312)	Non-painful TMD (*n* = 160)	Painful TMD (*n* = 390)
Gender			
Female (%)	201 (64.4)	106 (66.3)	304 (77.9)
Male (%)	111 (35.6)	54 (33.7)	86 (22.1)
Age (years) Mean (SD)	29.2 (9.00)	27.1 (7.87)	30.7 (9.46)
PVAQ scores Mean (SD)	31.3 (18.6)	35.3 (18.8)	45.1 (15.8)
TSK/TMD scores Mean (SD)	16.5 (5.80)	24.5 (7.50)	28.7 (7.68)

SD – Standard deviation; TMD **–** Temporomandibular joint disorders; TSK-TMD **-** Tampa Scale for Kinesiophobia for Temporomandibular Disorders; PVQA **-** Pain Vigilance and Awareness Questionnaire.

### Factor analysis of mixed data

#### All participants

No outliers were detected based on Mahalanobis distance. The eigenvalues for the first three dimensions were 2.03, 1.12, and 1.05, respectively. Since remaining dimensions presented eigenvalues below 1.0, only the first three dimensions were retained. These dimensions explained 33.9%, 18.7%, and 17.6% of the total variance, respectively, accounting for 70.2% of the overall variability. The contributions of each variable to the retained dimensions are presented in the Supplementary Material 1 and 2.

#### TMD subgroups

One outlier was detected based on Mahalanobis distance and removed from analysis. The eigenvalues for the first three dimensions were 2.34, 1.26, and 1.06, respectively, whilst the remaining dimensions presented eigenvalues below 1.0. The three retained dimensions explained 33.4%, 17.9%, and 15.1% of the total variance, accounting for 66.5% of the overall variability. The contributions of each variable for dimensions can be found in Supplementary Material 3 and 4.

### Cluster analysis

#### All participants

**Fig. 1:**
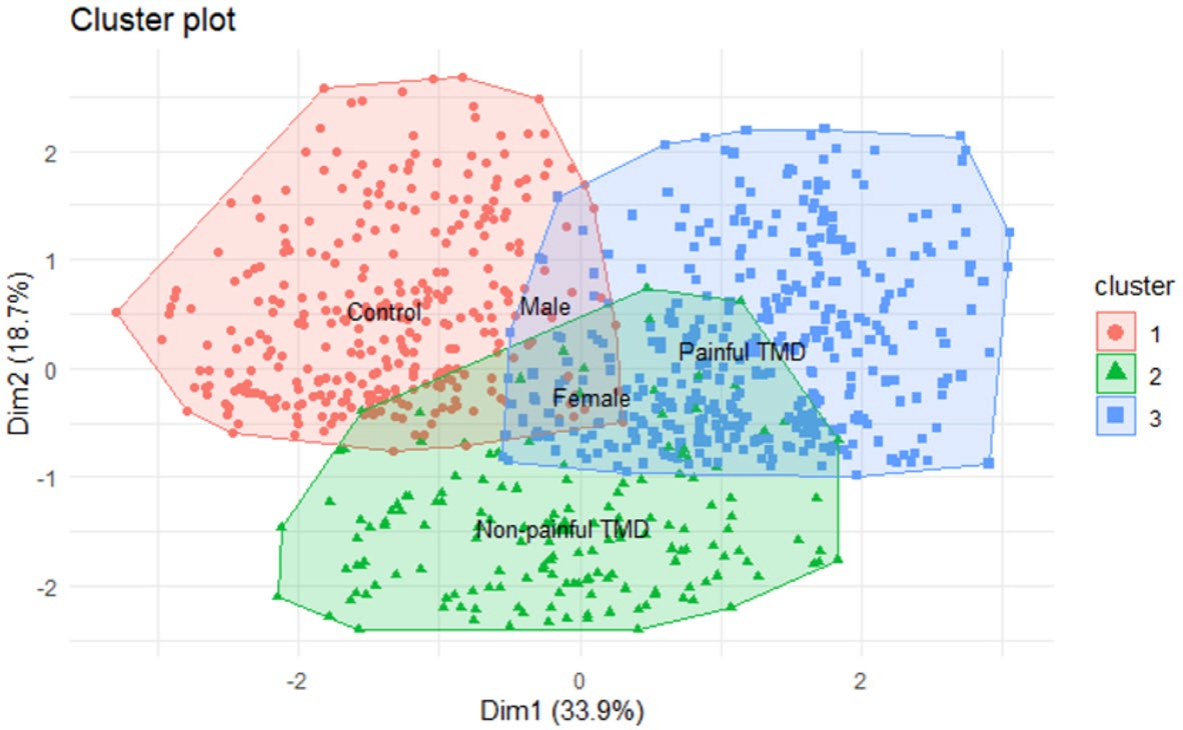
Hierarchical clustering on principal components. Cluster plot based on the complete dataset, including control subjects, non-painful TMD and painful TMD cases.

Based on the three retained dimensions from factor analysis, three clusters with distinct clinical and psychological profiles were identified. Cluster 1 consisted predominantly of controls (95%) and was characterized by a higher proportion of females and lower TSK/TMD and PVAQ scores. Cluster 2 included exclusively individuals with non-painful TMD and presented intermediate levels of TSK/TMD and PVAQ. Cluster 3 was composed mainly of individuals with painful TMD (98%), also with a predominance of females, and showed the highest TSK/TMD and PVAQ scores, consistent with a profile of greater psychological burden (Fig. 1; Table 2). Bootstrap analysis using Jaccard similarity coefficients showed good cluster stability (cluster 1 = 0.86, cluster 2 = 0.97, and cluster 3 = 0.78), indicating that clusters are stable and reproducible. Moreover, silhouette analysis showed an average silhouette width of 0.35 (cluster 1 = 0.32, cluster 2 = 0.35, and cluster 3 = 0.37), indicating acceptable cluster separation, with moderate structural overlap expected given the clinical continuity between groups.

**Table 2:** Characteristics of the data-driven clusters identified in the complete dataset, including control subjects, non-painful TMD, and painful TMD cases.

Cluster	Characteristics	Summary
Cluster 1	Predominantly controls (95%) More female (64%) Lower PVAQ scores (median: 33) Lower TSK/TMD scores (median: 14)	Controls
Cluster 2	All non-painful TMD (100%) Intermediate PVAQ scores (median: 37) Intermediate TSK/TMD scores (median: 24)	Non-painful TMD
Cluster 3	Predominantly painful TMD (98%) Mostly female (77%) Higher PVAQ scores (median: 48) Higher TSK/TMD scores (median 30)	Painful TMD/High psychological phenotype

TMD **–** Temporomandibular joint disorders; TSK/TMD **-** Tampa Scale for Kinesiophobia for Temporomandibular Disorders; PVQA **-** Pain Vigilance and Awareness Questionnaire.

#### TMD subgroups

**Fig. 2:**
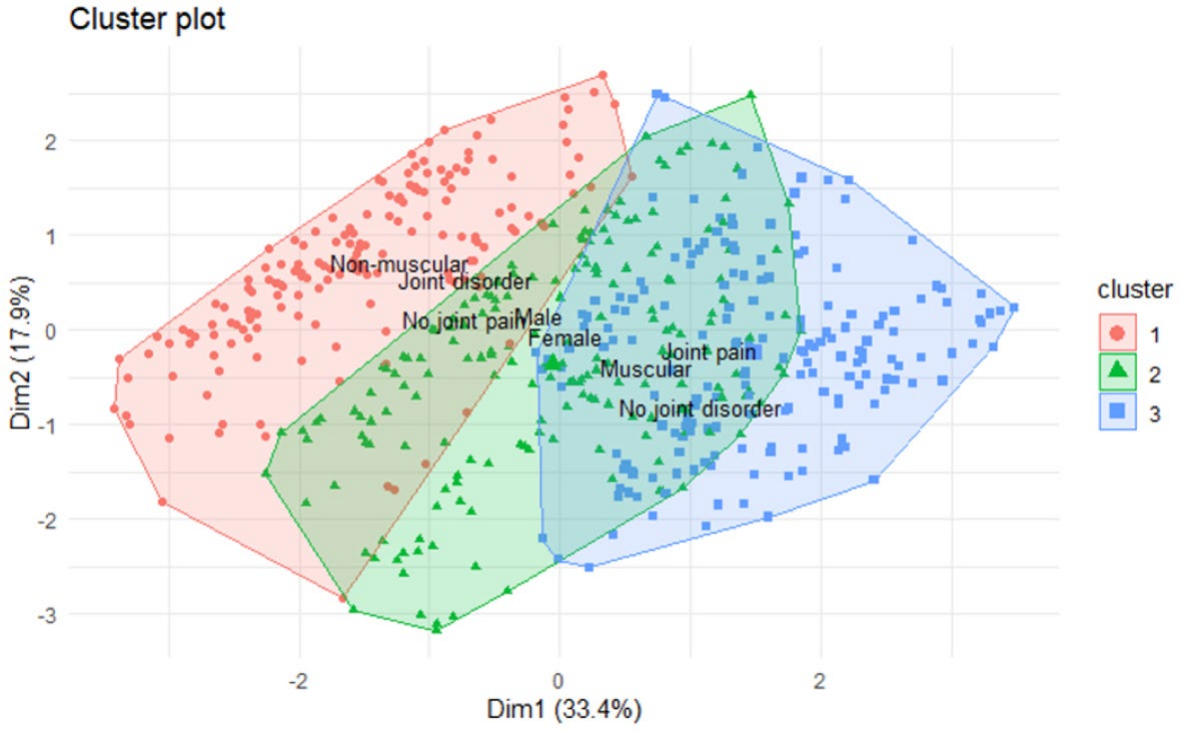
Hierarchical clustering on principal components. Cluster plot based on the TMD-only dataset, including joint pain, joint disorders, and muscular TMD cases.

Cluster analysis identified three overlapping clusters. Cluster 1 was predominantly characterized by joint disorders without pain, no myogenous diagnosis, younger individuals and low TSK/TMD and PVAQ scores. Cluster 2 consisted of myogenous TMD individuals, mostly no joint pain and low-to-moderate TSK/TMD and PVAQ scores. Cluster 3 was mostly characterized by joint pain combined with muscular TMD, moderate joint disorders, relatively older individuals and presenting higher TSK/TMD and PVAQ scores (Fig. 2; Table 3). Bootstrap analysis using Jaccard similarity coefficients showed an overall poorer cluster stability (cluster 1 = 0.61, cluster 2 = 0.49, and cluster 3 = 0.87) compared to the complete dataset. Whilst cluster 3 was highly stable, cluster 1 and 2 proved unstable. This indicates that the reduced sample size, coupled with the complexity of differences within TMD diagnoses, limited the model’s ability to establish distinct clinical profiles. Additionally, silhouette analysis demonstrated an average silhouette width of 0.20 (cluster 1 = 0.23, cluster 2 = 0.19, cluster 3 = 0.19), suggesting limited internal cohesion and separation between groups.

**Table 3:** Characteristics of the data-driven clusters identified in the TMD-only dataset (temporomandibular joint disorder subgroups).

Cluster	Characteristics	Summary
Cluster 1	Predominantly joint disorders (98%) Predominantly non-muscular (98%) Mostly no joint pain (84%) Slightly more female (67%) Younger individuals (median: 24) Lowest PVAQ scores (median 39.1) Lowest TSK/TMD scores (median 23.5)	Joint disorder without pain Low psychological profile
Cluster 2	All muscular TMD (100%) Mostly no joint pain (98%) Slightly higher PVAQ scores (median 44)	Muscular TMD with mixed joint disorders Low-moderate psychological profile
Cluster 3	Predominantly joint pain (98%) Mostly muscular TMD (86%) Mostly no joint disorder (70%) Older individuals Highest PVAQ scores (median 51) Highest TSK/TMD scores (median 31)	Joint pain with mixed muscular TMD High psychosocial profile

TMD **–** Temporomandibular joint disorders; TSK-TMD **-** Tampa Scale for Kinesiophobia for Temporomandibular Disorders; PVQA **-** Pain Vigilance and Awareness Questionnaire.

### Partial least squares discriminant analysis

#### All participants

**Fig. 3:**
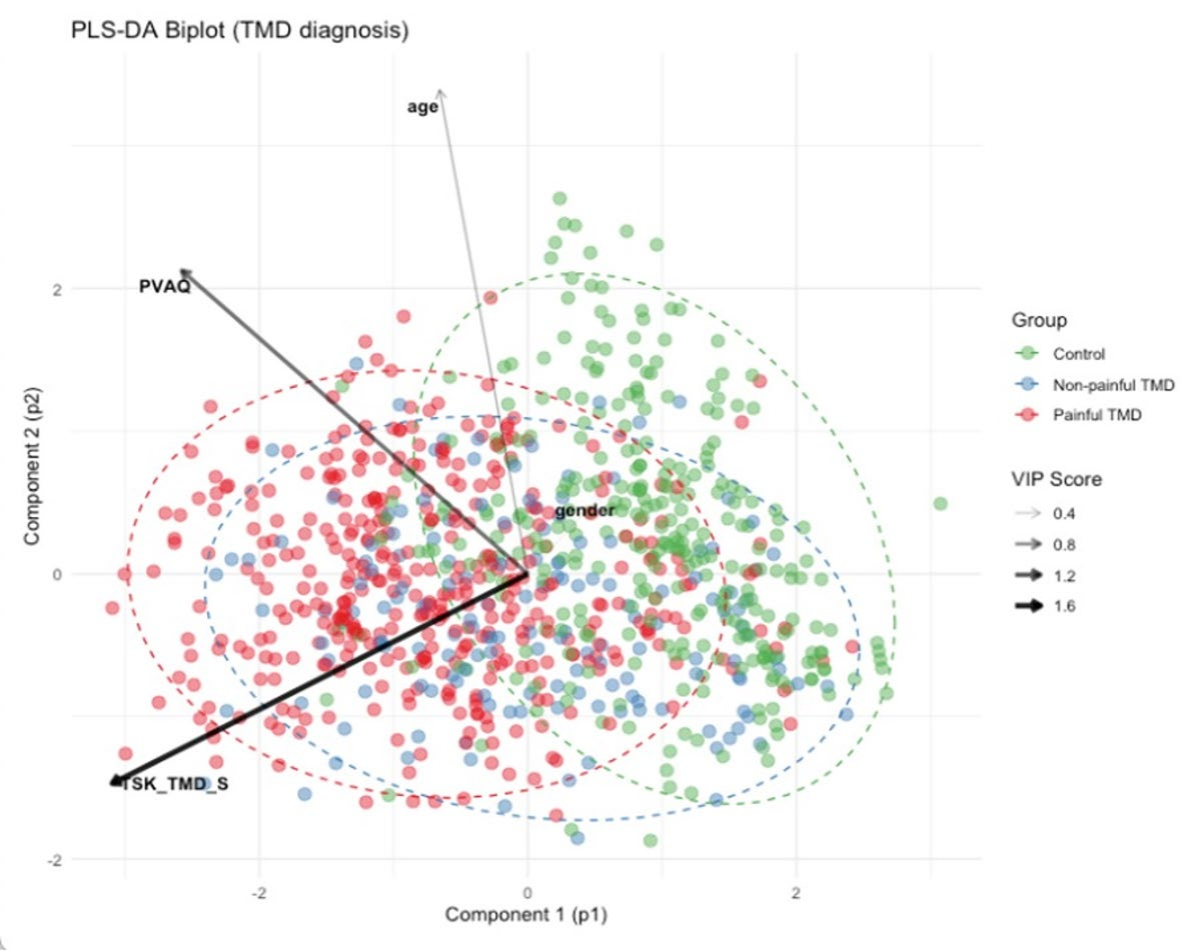
Biplot of partial least squares discriminant analysis components and VIP scores for controls, non-painful TMD, and painful TMD. Distribution of controls, non-painful TMD, and painful TMD across the first two partial least squares discriminant analysis components. Vectors indicate the direction and contribution of the variables included in the model, and arrow thickness reflects variable importance in projection (VIP) scores. Partial overlap among groups was observed.

The partial least squares discriminant analysis using the first two components showed significant discrimination between groups (pR2Y = 0.05; pQ2 = 0.05). However, the model showed modest explanatory and predictive performance (R2Y = 0.229; Q2 = 0.225). The model explained 55.2% of the variance in the predictor variables (R2X = 0.552), with a root mean square error of estimation (RMSEE) of 0.396. The small difference between R2Y and Q2 suggested minimal overfitting and acceptable model generalizability despite the modest overall performance (Supplementary Material 5). The VIP scores showed that TSK/TMD (VIP = 1.62) and PVAQ (VIP = 1.02) were the main contributors to group discrimination, whereas age (VIP = 0.47) and gender (VIP = 0.35) showed lower contributions (Supplementary Material 6). The biplot of the first two components and VIP scores indicated that painful TMD was more closely related to higher TSK/TMD and PVAQ values. Controls were positioned farther from these variables, whereas non-painful TMD occupied an intermediate position with partial overlap with both groups (Fig. 3).

### TMD subgroups

**Fig. 4:**
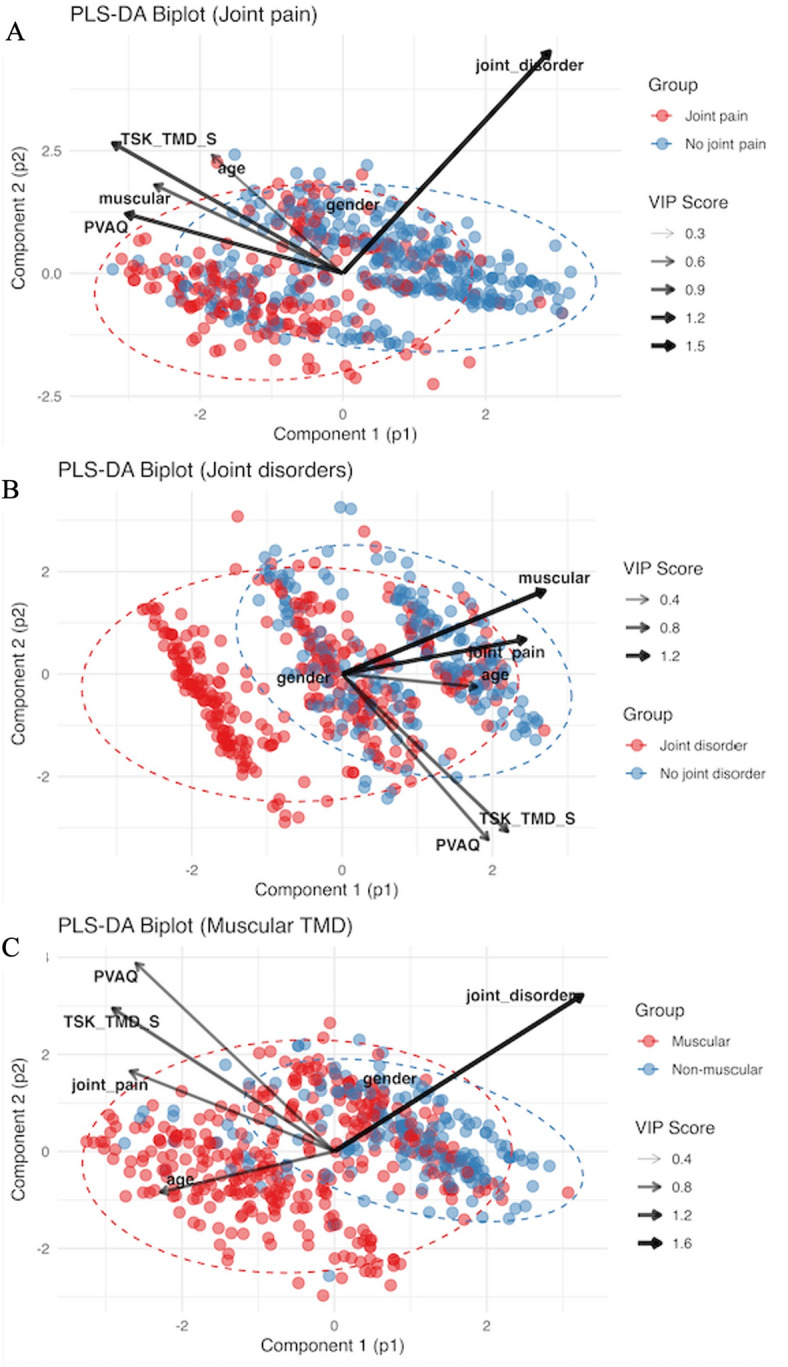
Biplot of partial least squares discriminant analysis components and VIP scores for the TMD subgroups, including joint pain, joint disorders, and muscular TMD cases. (**A**) Joint pain, (**B**) joint disorders, and (**C**) muscular TMD. The biplots show the distribution of the subgroups across the first two partial least squares discriminant analysis components. Vectors indicate the direction and contribution of the variables included in each model. Partial overlap among groups was observed.

Partial Least Squares Discriminant Analysis performed on the first two components showed statistically significant discrimination among the diagnostic subgroups (*p* = 0.05), although the explained variance and predictive performance were modest. For joint pain R2Y and Q2 were 0.199 and 0.188, 0.272 and 0.245 for joint disorders, and for muscular TMD, 0.242 and 0.230. The small differences between R2Y and Q2 suggested limited overfitting and acceptable model generalizability, although overlap among subgroups remained (Supplementary Material 7). VIP analysis indicated that TSK/TMD (VIP = 1.08) and PVAQ (VIP = 1.15) contributed the most to the discrimination of joint pain and were the principal contributors just for this diagnosis. Age and gender showed lower contributions in all analyses (Supplementary Material 8). Therefore, the biplot of the first two components and VIP scores indicated that joint pain was more closely related to higher kinesiophobia and hypervigilance, whereas joint disorders and muscular TMD were more strongly related to structural diagnostic variables (Fig. 4).

### Multivariate association analyses

#### All participants

The MANCOVA model (Table 4) revealed a significant multivariate effect of diagnostic group (*p* < 0.001), indicating overall differences in the combined TSK/TMD and PVAQ profiles across controls, non-painful TMD, and painful TMD participants. Age also showed a significant multivariate effect (*p* = 0.001), whilst gender was not statistically significant (*p* = 0.125). Furthermore, the diagnostic group × age interaction was statistically significant (*p* < 0.001), indicating that the association between age and the combined outcome profile differed across the three diagnostic groups.

**Table 4:** Multivariate analysis of covariance (MANCOVA) of TSK/TMD and PVAQ scores: diagnostic group differences and the diagnostic group × age interaction, with age and gender as covariates.

	Pillai’s Trace*	Approx F (df1, df2)	*p*-value
Diagnostic groups (target)	0.404	108.300 (4, 1710)	*p* < 0.001
Gender (covariate)	0.005	2.100 (2, 854)	*p* = 0.126
Age (covariate)	0.016	6.900 (2, 854)	*p* = 0.001
Diagnostic group x age (interaction)	0.026	5.700 (4, 1710)	*p* < 0.001

df1 – numerator degrees of freedom; df2 – denominator degrees of freedom. *Type III multivariate tests (Pillai’s trace), with sum-to-zero contrasts for factors and age mean-centred. A diagnostic group × gender interaction was tested but was non-significant and therefore excluded from the final model.

Follow-up univariate ANCOVA models (Supplementary Material 9) indicated that, for PVAQ scores, the diagnostic group × age interaction was not significant (*p* = 0.419), and only the main effect of diagnostic group reached significance (*p* < 0.001). Pairwise comparisons (Supplementary Material 10) showed that all three diagnostic groups differed significantly on PVAQ (all *p* < 0.05), with lowest scores in controls and highest in painful TMD participants.

For TSK/TMD scores, there was a significant diagnostic group × age interaction (*p* = 0.002). Simple slope analyses (Supplementary Material 11–12) showed that age was not significantly associated with TSK/TMD scores in the control group (*p* = 0.510), whereas significant positive associations were observed in both the non-painful (*p* = 0.006) and painful TMD groups (*p* < 0.001), indicating that higher age was associated with greater TSK/TMD scores among participants with TMD. Pairwise comparisons further indicated that controls differed significantly from both TMD groups at any age, whilst the two TMD groups significantly differed from each other up to approximately 45 years of age (Supplementary Material 13).

#### TMD subgroups

The multivariate model revealed significant associations for age (*p* = 0.001), gender (*p* = 0.022), PVAQ (*p* = 0.004), and TSK/TMD (*p* = 0.001), and (Table 4). Follow-up outcome-specific univariate analyses, adjusted for multiple comparisons, revealed distinct association patterns across variables. Age was significantly associated with all TMD subgroups, including joint pain (*p* = 0.016), joint disorders (*p* = 0.002), and muscular TMD (*p* = 0.001). Gender (*p* = 0.031) and TSK/TMD (*p* = 0.001) showed subgroup-specific patterns, with significant associations linked to muscular TMD, and no observable relationship to joint-related outcomes. PVAQ demonstrated an isolated association with joint pain (*p* = 0.001), with no significant associations observed for joint disorders (*p* = 0.598) or muscular TMD (*p* = 0.598).

### Multinomial logistic regression analysis

Findings from the multinomial logistic regression examining variables associated with the likelihood of a TMD diagnosis relative to controls are provided in Table 5. The regression model presented good discrimination for control (AUC = 0.87) and painful TMD (AUC = 0.83), with acceptable overall accuracy (67.4%) and good calibration across all three classes (Brier scores: control = 0.14, non-painful TMD = 0.14, painful TMD = 0.17). Discrimination for non-painful TMD was limited (AUC = 0.68), suggesting that the study variables might be insufficient to characterize this group, in particular.

**Table 5:** Associations between psychosocial factors and TMD-only subgroups using a multivariate generalized linear model.

	Multivariate Wald (all TMD subgroups)		Follow-up univariate Wald (per TMD subgroup)		
	W statistic	*p*-value	Joint Pain (*p*-values)	Joint Disorder (*p*-values)	Muscular (*p*-values)
Global model	W = 11.68	< 0.001	**–**	**–**	**–**
Age	W = 7.93	< 0.001	0.016	0.002	0.001
Gender	W = 3.11	0.027	0.215	0.573	0.031
PVAQ	W = 3.96	0.002	0.001	0.598	0.598
TSK/TMD	W = 7.73	< 0.001	0.311	0.292	0.001

TSK-TMD - Tampa Scale for Kinesiophobia for Temporomandibular Disorders; PVAQ - Pain Vigilance and Awareness Questionnaire; PIT-trap resampling was used to account for correlations between outcome variables; *p-values are adjusted for multiple comparisons using step-down resampling;

Nonetheless, higher TSK/TMD scores were significantly associated with both non-painful (OR = 7.94, *p* < 0.001) and painful TMD (OR = 11.46, *p* < 0.001). PVAQ demonstrated an inverse association with the non-painful TMD group (OR = 0.79, *p* < 0.001). Lastly, males presented an inverse relationship with painful TMD (OR = *p* < 0.001), suggesting that females are more likely to experience painful TMD.

## Discussion

The present multicenter study examined the relationship between TMD diagnoses, pain-related hypervigilance, and kinesiophobia in a large sample. The findings indicate that these cognitive-behavioral factors were primarily associated with the presence of pain and complex TMD presentations, rather than with joint disorders alone. Individuals with painful TMD consistently exhibited higher levels of hypervigilance and kinesiophobia compared to controls and with individuals with non-painful TMD. Furthermore, cluster and multivariate analyses revealed distinct profiles, in which complex TMD presentations including mainly painful diagnosis and in lower proportion non-painful diagnosis (joint disorders) were accompanied by higher levels of these cognitive-behavioral variables. Together, these results suggest that hypervigilance and kinesiophobia may contribute to the differentiation of different TMD diagnosis and the presentation of complex cases.

In musculoskeletal pain conditions, cognitive processes such as attentional bias toward pain and fear of movement have been associated with greater symptom severity and functional limitations^27,28^. In this context, hypervigilance and kinesiophobia may represent complementary mechanisms through which cognitive factors influence pain-related behavior^13,29^. Experimental and clinical studies have shown that individuals with persistent pain often display attentional biases toward pain-related stimuli and greater difficulty disengaging from pain cues, which may increase awareness of bodily sensations and reinforce symptom monitoring^3,7,30^. Consistent with these mechanisms, the present study found that hypervigilance was closely associated with pain status in TMD. Participants with painful TMD exhibited higher PVAQ scores than both controls and individuals with non-painful TMD. This pattern suggests that hypervigilance may be more closely related to pain diagnoses than to joint disorders alone. Similar associations between increased pain vigilance, symptom severity, and pain-related disability have been reported in chronic orofacial pain populations, supporting the idea that hypervigilance alone does not necessarily cause or raises pain, but sustained attention to bodily or somatic sensations may contribute to the amplification and persistence of pain experiences^4,7,13^.

Within the fear-avoidance framework, individuals who interpret pain as threatening may develop fear of movement, leading to behavioral strategies aimed at limiting activities perceived as potentially painful^10,11^. Although such responses may initially serve a protective function, persistent avoidance can contribute to reduced function and maintenance of pain-related disability^10,11^. In TMD, fear of jaw movement has been associated with greater functional limitation and interference with daily activities^31,15^. The present findings are consistent with this framework, as kinesiophobia showed a clear gradient across groups: TSK/TMD scores were lowest in controls, intermediate in individuals with non-painful TMD, and highest in participants with painful TMD. This pattern suggests that fear of movement is also closely linked to TMD pain status. Kinesiophobia also differed across diagnostic groups depending on age. Older participants presented with higher kinesiophobia scores among participants with TMD, in both the painful and non-painful subtypes, but not among controls. From a pathophysiological perspective, heightened fear of movement may interact with nociceptive processing through several mechanisms. Increased anticipation of pain may enhance attention to mandibular sensations and promote protective motor behaviors, such as reduced jaw movement amplitude or altered muscle recruitment, which could contribute to increased muscular loading and symptom persistence^10,13,31^. In addition, fear-related responses have been associated with changes in central pain modulation and increased responsiveness to nociceptive input in chronic pain conditions^33,34^.

Consistent with previous observations, the present study identified a strong association between kinesiophobia and hypervigilance, suggesting that fear of movement and heightened attention to pain may represent interconnected components of pain-related cognitive processing in TMD populations^13,29^. One possible explanation is that the activation of the fear system of pain increases vigilance toward bodily signals and promotes behavioral strategies aimed at avoiding pain. These avoidance responses may progressively reinforce fear of movement, which can sustain negative expectations about pain and increase sensitivity to somatic cues. As a result, individuals may become more attentive to bodily sensations, further strengthening hypervigilant monitoring of pain^7,29^. Similar interactions between fear-related avoidance and increased attention to pain have been reported in other chronic pain conditions, including low back pain and fibromyalgia, where these processes have been linked to greater symptom persistence, increased pain sensitivity, and functional limitations^10,19,29^. Therefore, the association between kinesiophobia and hypervigilance seems to be bidirectional. However, even though our regression model indicated higher odds for kinesiophobia for painful and non-painful TMD, PVAQ scores presented lower odds just in non-painful TMD when considered relative to the remaining variables, indicating that these individuals presented a disproportionally low hypervigilance compared to controls when also considering their high kinesiophobia. This finding may suggest that elevated kinesiophobia can be present even in the absence of pronounced pain vigilance and independently of the diagnosis, showing a probable major role of kinesiophobia in TMD patients.

The cluster analyses provide additional insight into the relationship between these cognitive-behavioral factors and clinical presentations of TMD. When considering controls and TMD participants together, three data-driven clusters emerged that broadly corresponded to different clinical and cognitive-behavioral profiles. The first one was dominated by controls and was characterized by low levels of both hypervigilance and kinesiophobia. The second one consisted exclusively of individuals with non-painful TMD and showed intermediate values of these variables. In contrast, the third data-driven cluster included primarily individuals with painful TMD and exhibited the highest levels of hypervigilance and kinesiophobia. These findings suggest that pain-related cognitive-behavioral responses may differentiate TMD presentation patterns beyond the mere presence of joint disorders. When the analysis focused just on TMD subgroups, three exploratory profiles were identified; however, bootstrap stability and silhouette analysis indicated poor reproducibility and limited internal cohesion, suggesting substantial overlap between groups. Data-driven clusters 1 and 2, which fell below the pre-defined stability threshold, suggested broad tendencies only: data-driven cluster 1 appeared to consist mainly of joint disorders without pain and low levels of hypervigilance and kinesiophobia, while data-driven cluster 2 appear to consist mainly of muscular TMD with modest cognitive-behavioral involvement. On the other hand, data-driven cluster 3 was the only profile demonstrating acceptable stability and included individuals with joint pain, muscular diagnoses and in less proportion with joint disorders, with higher levels of both hypervigilance and kinesiophobia. These results suggest that TMD presentations involving pain and multiple diagnostic components may be associated with greater psychological burden. Importantly, these data-driven clusters were supported by multivariate analyses which indicated that hypervigilance and kinesiophobia were the strongest contributors to the discrimination of joint pain. This may suggest that these cognitive-behavioral variables may play a greater role in differentiating painful TMD diagnoses than joint disorders diagnoses alone. Although the predictive performance of the models was modest, the consistent association between these variables and pain-related phenotypes supports the relevance of cognitive-behavioral factors in TMD (Table 6).

**Table 6:** Multinomial logistic regression results for TMD diagnostic groups, with controls as the reference category.

Variables	Non-painful TMD vs. reference (control)		Painful TMD vs. reference (control)	
	OR (95% CI)	*p*-value	OR (95% CI)	*p*-value
Age	0.96 (0.93–0.98)	< 0.010	1.00 (0.98–1.02)	0.976
Male gender (reference female)	0.74 (0.47–1.18)	0.203	0.34 (0.22–0.53)	< 0.001
PVAQ*	0.79 (0.69–0.90)	< 0.001	1.01 (0.89–1.14)	0.905
TSK/TMD*	7.94 (5.43–11.59)	< 0.001	11.46 (8.01–16.25)	< 0.001

OR - Odds Ratio; CI - Confidence Interval; * OR per 10-unit increase.

Traditional diagnostic frameworks often focus on psychological factors like depression, somatization and anxiety which can influence symptom severity, treatment response, and long-term outcomes^34–36^. However, the present findings highlight the additional importance of considering hypervigilance and kinesiophobia when evaluating patients with TMD. Screening for cognitive-behavioral factors may therefore provide valuable information for identifying patients at greater risk of persistent pain or disability. According to our results, these two factors should be assessed especially in patients with painful TMD diagnosis or with multiple painful and non-painful diagnoses, since both variables showed the strongest contribution to group discrimination. The assessment of these factors could allow to propose interventions targeting maladaptive pain-related beliefs and attentional patterns—such as cognitive behavioral therapy (thought monitoring), pain education, and graded exposure approaches— which have demonstrated benefits in several other chronic pain conditions and may also be relevant for patients with painful TMD^6,37,38^. In addition, a prospective case series study using a biobehavioral multimodal physiotherapy approach in patients with chronic craniofacial pain reported improvements in pain intensity, disability, and psychosocial variables, supporting the relevance of targeting cognitive-behavioral factors in this population^39^. In line with these findings, randomized controlled trials in TMD populations have shown that adding pain education and therapeutic exercise to conventional care, can improve outcomes related to kinesiophobia and disability compared with standard approaches alone^40,41^.

The strengths of this study include the large sample size, the multicenter design involving three countries, and the comprehensive analytical approach combining traditional statistical methods with multivariate and clustering techniques. The inclusion of both painful and non-painful TMD presentations allowed for a more detailed examination of how cognitive-behavioral variables relate to different clinical profiles. Furthermore, the use of validated instruments to assess hypervigilance and kinesiophobia enhances the reliability of the findings. However, several limitations should also be considered. First, the cross-sectional design precludes conclusions about causal relationships between psychosocial variables and pain. It remains unclear whether hypervigilance and kinesiophobia contribute to the development of painful TMD or emerge as a consequence of persistent pain. Longitudinal studies are needed to clarify the temporal relationships between these factors. Second, although the multicenter design enhances generalizability, cultural or contextual differences between countries may influence cognitive-behavioral responses to pain and limit direct comparability across sites. Third, because individuals with previous or ongoing treatment for TMD/orofacial pain and other conditions that could influence pain perception were excluded during pre-screening, the findings cannot be extrapolated to these populations. Fourth, the predictive performance of the multivariate models was modest, indicating that additional biological, behavioral, or environmental factors likely contribute to the observed clinical variability. Finally, the study focused on selected cognitive-behavioral constructs, and other factors such as catastrophizing and coping strategies may also play important roles in TMD phenotypes. Future research should aim to further characterize psychosocial phenotypes in TMD using longitudinal and mechanistic approaches. Integrating psychological, neurobiological, and behavioral data may help clarify how cognitive processes interact with nociceptive mechanisms in the development and persistence of TMD pain. Such approaches may also contribute to more personalized treatment strategies that address both the biological and psychosocial dimensions of the disorder.

## Conclusions

Hypervigilance and kinesiophobia were primarily associated with painful TMD diagnosis rather than with joint disorders alone. Individuals presenting combined or more clinically complex painful TMD conditions consistently exhibited higher levels of these cognitive-behavioral factors. Cluster-based and multivariate analyses suggested potentially distinct exploratory data-driven profiles of TMD, characterized by different pain presentations and psychosocial burden. Profiles involving joint pain - whether alone or in combination with muscular diagnoses- were associated with increased hypervigilance and kinesiophobia, whereas isolated non-painful joint conditions showed comparatively lower levels of these variables. These findings support the existence of associations between cognitive-behavioral factors and distinct TMD clinical presentations, while reinforcing the importance of considering psychosocial dimensions when characterizing painful and clinically complex TMD conditions.

## Supplementary Information

Below is the link to the electronic supplementary material.

Supplementary Material 1

## Data Availability

The datasets used and/or analyzed during the current study are available from the corresponding author on reasonable request.
