# Supplementary material for: Hypervigilance and kinesiophobia characterize distinct exploratory data-driven profiles of temporomandibular disorders
Source: Sci Rep. 2026 Jun 29;16:19786. doi: 10.1038/s41598-026-60271-w (PMC13315795; doi:10.1038/s41598-026-60271-w)
Supplement: Supplementary file 1 — Supplementary Material 1 [file 41598_2026_60271_MOESM1_ESM.docx]

**Supplementary Material 1. Factor analysis of mixed data. Variable contribution to retained dimensions for the complete dataset, including controls, non-painful TMD, and painful TMD cases.**

| **Variables** | **Dimension 1** | **Dimension 2** | **Dimension 3** |
| --- | --- | --- | --- |
| **Age** | 2.31% | 47.25% | 0.99% |
| **PVAQ** | 26.87% | 0.36% | 0.32% |
| **TSK/TMD** | 38.86% | 0.48% | 1.60% |
| **Female** | 0.17% | 3.24% | 19.04% |
| **Male** | 0.40% | 7.89% | 46.36% |
| **Control** | 16.75% | 8.43% | 0.95% |
| **Non-painful TMD** | 0.06% | 31.36% | 25.24% |
| **Painful TMD** | 14.58% | 0.98% | 5.51% |

**Legend:** TMD – Temporomandibular joint disorders; TSK-TMD - Tampa Scale for Kinesiophobia for Temporomandibular Disorders; PVQA - Pain Vigilance and Awareness Questionnaire.

Supplementary Material 2. Scree plot showing the proportion of variance explained by each dimension derived from factor analysis of mixed data for complete dataset**, including controls, non-painful TMD, and painful TMD cases.**

**
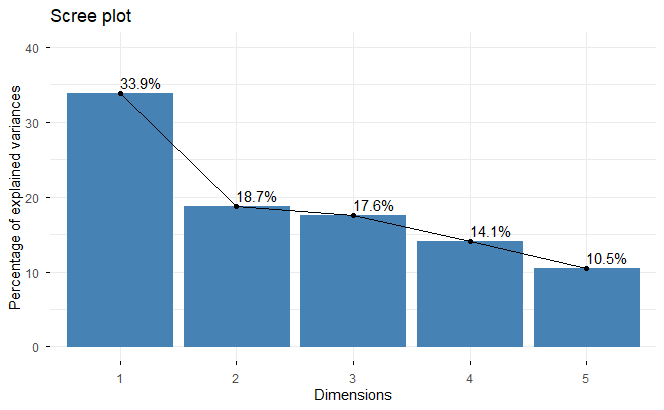
**

Supplementary Material 3. **Factor analysis of mixed data. Variable contribution to retained dimensions for the TMD-only dataset, including joint pain, joint disorders, and muscular TMD cases.**

| **Variables** | **Dimension 1** | **Dimension 2** | **Dimension 3** |
| --- | --- | --- | --- |
| **Age** | 8,52% | 4,69% | 21,1% |
| **PVAQ** | 21,94% | 27,87% | 0,31% |
| **TSK/TMD** | 25,04% | 20,48% | 0,14% |
| **Female** | 0,04% | 0,13% | 19,14% |
| **Male** | 0,11% | 0,39% | 55,91% |
| **Joint pain** | 9,38% | 0,98% | 1,94% |
| **No joint pain** | 5,94% | 0,62% | 1,23% |
| **Joint disorder** | 5,69% | 10,87% | 0,01% |
| **No joint disorder** | 8,51% | 16,25% | 0,01% |
| **Muscular TMD** | 5,21% | 6,23% | 0,07% |
| **Non-muscular TMD** | 9,62% | 11,49% | 0,13% |

**Legend:** TMD – Temporomandibular joint disorders; TSK-TMD - Tampa Scale for Kinesiophobia for Temporomandibular Disorders; PVQA - Pain Vigilance and Awareness Questionnaire.

Supplementary Material 4. Scree plot showing the proportion of variance explained by each dimension derived from factor analysis of mixed data for **TMD-only dataset, including joint pain, joint disorders, and muscular TMD cases.**

**
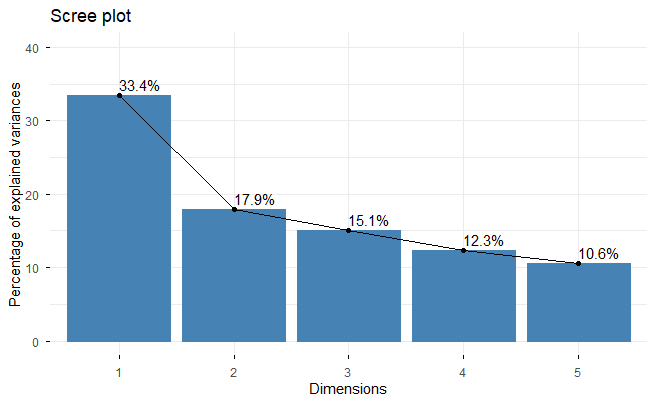
**

Supplementary Material 5. Partial least squares discriminant analysis model performance for discrimination between control subjects, non-painful temporomandibular disorders, and painful temporomandibular disorders.

| **Group** | **R2X**  **(cum)** | **R2Y**  **(cum)** | **Q2**  **(cum)** | **RMSEE** | **pre** | **ort** | **pR2Y** | **pQ2** |
| --- | --- | --- | --- | --- | --- | --- | --- | --- |
| **Diagnosis** | 0.552 | 0.229 | 0.225 | 0.396 | 2 | 0 | 0.05 | 0.05 |

R2X(cum) - Cumulative proportion of variance in the X variables (predictors) explained by the model; R2Y (cum) - Cumulative proportion of variance in the Y variable (class labels) explained; Q2 (cum) - Cumulative predictive ability, estimated by cross-validation; RMSEE - Root Mean Square Error of Estimation. Error of the model on the training data; pre - Number of components that explain variation in X relevant to Y; ort - Orthogonal components. Components that explain variation in X unrelated to Y; pR2Y - p-value for R2Y from permutation testing; pQ2 - p-value for Q2 from permutation testing.

Supplementary Material 6. Variable importance in projection (VIP) for discrimination among control subjects, non-painful temporomandibular disorders, and painful temporomandibular disorders.

| **Variables** | **Diagnosis** |
| --- | --- |
| **Age** | 0.47 |
| **Gender** | 0.35 |
| **TSK-TMD-S** | 1.62 |
| **PVAQ** | 1.02 |

**Legend:** TMD – Temporomandibular joint disorders; TSK-TMD - Tampa Scale for Kinesiophobia for Temporomandibular Disorders; PVQA - Pain Vigilance and Awareness Questionnaire.

Supplementary Material 7. Partial least squares discriminant analysis (PLS-DA) model performance for discrimination among temporomandibular disorder subgroups.

| **Group** | **R2X (cum)** | **R2Y (cum)** | **Q2 (cum)** | **RMSEE** | **pre** | **ort** | **pR2Y** | **pQ2** |
| --- | --- | --- | --- | --- | --- | --- | --- | --- |
| **Joint pain** | 0.471 | 0.199 | 0.188 | 0.437 | 2 | 0 | 0.05 | 0.05 |
| **Joint disorder** | 0.512 | 0.272 | 0.245 | 0.419 | 2 | 0 | 0.05 | 0.05 |
| **Muscular** | 0.510 | 0.242 | 0.230 | 0.417 | 2 | 0 | 0.05 | 0.05 |

R2X(cum) - Cumulative proportion of variance in the X variables (predictors) explained by the model; R2Y (cum) - Cumulative proportion of variance in the Y variable (class labels) explained; Q2 (cum) - Cumulative predictive ability, estimated by cross-validation; RMSEE - Root Mean Square Error of Estimation. Error of the model on the training data; pre - Number of components that explain variation in X relevant to Y; ort - Orthogonal components. Components that explain variation in X unrelated to Y; pR2Y - p-value for R2Y from permutation testing; pQ2 - p-value for Q2 from permutation testing.

Supplementary Material 8. Variable importance in projection (VIP) scores for discrimination among temporomandibular joint disorders subgroups.

| **Variables** | **Joint pain** | **Joint disorder** | **Muscular** |
| --- | --- | --- | --- |
| **Age** | 0.60 | 0.73 | 0.90 |
| **Gender** | 0.28 | 0.11 | 0.37 |
| **Joint pain** | - | 1.31 | 0.83 |
| **Joint disorder** | 1.51 | - | 1.66 |
| **Muscular** | 0.86 | 1.57 | - |
| **TSK-TMD-S** | 1.08 | 0.82 | 0.97 |
| **PVAQ** | 1.15 | 0.74 | 0.80 |

**Legend:** TMD – Temporomandibular joint disorders; TSK-TMD - Tampa Scale for Kinesiophobia for Temporomandibular Disorders; PVQA - Pain Vigilance and Awareness Questionnaire.

Supplementary Material 9. Univariate Type III ANCOVA follow-up models of TSK/TMD and PVAQ scores across diagnostic groups, adjusted for gender, with mean-centred age and including diagnostic group × age interaction.

| **Outcome** | **Variable** | **F statistic (df1, df2)*** | **p-value** |
| --- | --- | --- | --- |
| PVAQ | Diagnostic group | 56.81 (2,855) | <0.001 |
|  | Age | 0.27 (1,855) | 0.648 |
|  | Gender | 1.73 (1,855) | 0.189 |
|  | Diagnostic group × Age | 0.82 (2,855) | 0.419 |
| TSK/TMD** | Diagnostic group | 265.94 (2,855) | <0.001 |
|  | Age | 12.18 (1,855) | <0.001 |
|  | Gender | 3.95 (1,855) | 0.047 |
|  | Diagnostic group × Age | 6.22 (2,855) | 0.002 |

**Legend:** TMD – Temporomandibular joint disorders; TSK-TMD - Tampa Scale for Kinesiophobia for Temporomandibular Disorders; PVQA - Pain Vigilance and Awareness Questionnaire. *Although Levene’s tests indicated heteroscedasticity in both outcomes, the large sample size renders ANCOVA F-tests reasonably robust to such violations. **Assumptions of homogeneity of regression slopes were violated for TSK/TMD, indicating moderation by age, whereas they were met for PVAQ.

Supplementary Material 10. Holm-adjusted pairwise contrasts between diagnostic groups (estimated marginal means) for TSK/TMD and PVAQ.

| **Outcome** | **Diagnostic group** | **Mean difference*** | **SE** | **t-value** | **p-value** |
| --- | --- | --- | --- | --- | --- |
| **PVAQ** | Control vs Non-painful TMD | −3.75 | 1.75 | −2.17 | 0.032 |
|  | Control vs Painful TMD | −13.92 | 1.34 | −10.38 | <0.001 |
|  | Non-painful TMD vs Painful TMD | −10.18 | 1.70 | −5.98 | <0.001 |
| **TSK/TMD**** | Control vs Non-painful TMD | −8.54 | 0.693 | −12.30 | <0.001 |
|  | Control vs Painful TMD | −12.19 | 0.533 | −22.87 | <0.001 |
|  | Non-painful TMD vs Painful TMD | −3.65 | 0.674 | −5.44 | <0.001 |

**Legend:** SE – Standard error; TMD – Temporomandibular joint disorders; TSK-TMD - Tampa Scale for Kinesiophobia for Temporomandibular Disorders; PVQA - Pain Vigilance and Awareness Questionnaire. *Mean difference equals to the mean first listed group minus the mean of the second listed group. **Given the significant interaction between diagnostic group and age for TSK/TMD, pairwise comparisons between groups must be interpreted conditionally across levels of age, and simple slope analyses were used to characterize age effects within each diagnostic group (Supplementary Material 12 and 13).

Supplementary Material 11. Simple slopes of age on TSK/TMD scores within each diagnostic group, decomposing the significant diagnostic group × age interaction.

| **Diagnostic group** | **Age slope*** | **SE** | **95%CI** | **t-value** | **p-value** |
| --- | --- | --- | --- | --- | --- |
| **TSK/TMD** | | | | | |
| Control | -0.029 | 0.044 | [-0.115, 0.057] | -0.659 | p = 0.510 |
| Non-painful TMD | 0.195 | 0.071 | [0.055, 0.329] | 2.737 | p = 0.006 |
| Painful TMD | 0.153 | 0.037 | [0.079, 0.225] | 4.090 | p < 0.001 |

**Legend:** SE – Standard error; 95%CI – 95% Confidence interval; TMD – Temporomandibular joint disorders; TSK-TMD - Tampa Scale for Kinesiophobia for Temporomandibular Disorders; PVQA - Pain Vigilance and Awareness Questionnaire. *Within-group slopes (unstandardized, per year of age) and 95% CIs estimated as marginal trends from the univariate Type III ANCOVA, with sum-to-zero contrasts and age mean-centred; estimates averaged over gender. Reported for TSK/TMD only, as the group × age interaction was non-significant for PVAQ.

Supplementary Material 12 - Association between age and TSK/TMD scores by diagnostic group, illustrating the significant diagnostic group × age interaction.


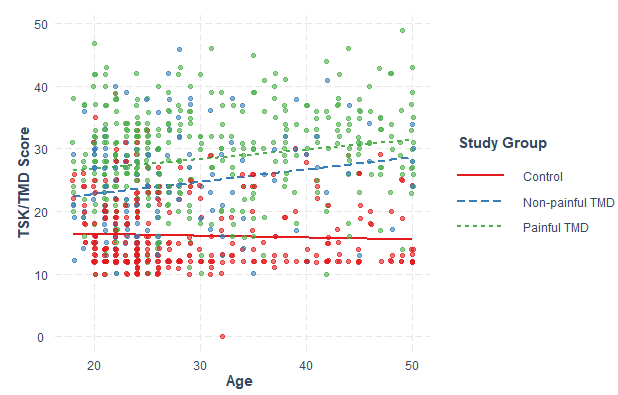


**Legend:** Lines represent model-implied (fitted) regressions of TSK/TMD on age within each diagnostic group, from a linear model including the diagnostic group × age interaction and adjusted for gender. Points represent observed scores; age is shown in years.

Supplementary Material 13 - Johnson–Neyman regions of significance for pairwise diagnostic-group differences in TSK/TMD across age.


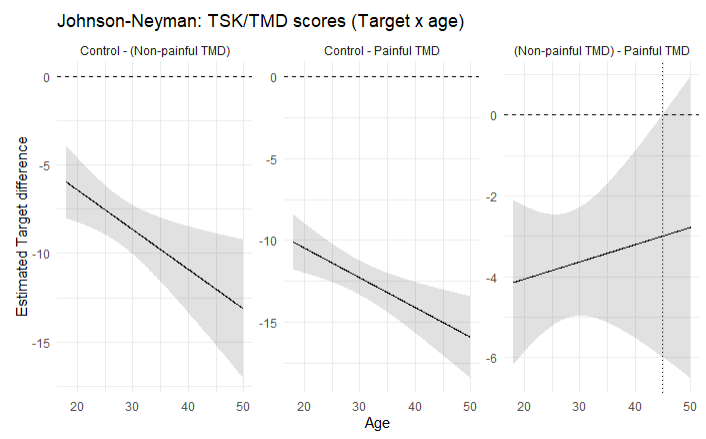


**Legend:** Estimated pairwise differences between diagnostic groups in TSK/TMD across the observed age range, derived from the univariate Type III ANCOVA (diagnostic group × age + gender; sum-to-zero contrasts; averaged over gender). Shaded bands are 95% CI. A difference is significant where the interval excludes zero. Control differed from both TMD groups across the entire observed age range, whereas the non-painful and painful TMD groups differed only below approximately 46 years.
